# Revealing the Atomic Structure of Blue Phosphorus Phases on Au(111) with Noncontact Atomic Force Microscopy

**DOI:** 10.1021/acsnano.6c03130

**Published:** 2026-06-08

**Authors:** Outhmane Chahib, Alberto Verdini, Zhenyu Li, Abdelkader Kara, Simone Del Puppo, Maria Peressi, Ernst Meyer, Rémy Pawlak

**Affiliations:** † Department of Physics, WSS Research Center for Molecular Quantum Systems, 243268 University of Basel, Klingelbergstrasse 82, Basel 4056, Switzerland; ‡ CNR—Instituto Officina dei Materiali (IOM), S.S. 14 km 163.5 in AREA Science Park, Trieste 34129, Italy; § State Key Laboratory of Precision and Intelligent Chemistry, 12652 University of Science and Technology of China, Hefei 230026, China; ∥ Department of Physics, 6243 University of Central Florida, Orlando, Florida 32816, United States; ⊥ Physics Department, 9315 University of Trieste, via A. Valerio 2, Trieste 34127, Italy

**Keywords:** blue-phosphorus, atomic structure, noncontact atomic force microscopy, force spectroscopy, density functional theory

## Abstract

Blue phosphorene (BlueP), a two-dimensional phosphorus allotrope with a buckled honeycomb lattice, has attracted significant interest for its semiconducting properties that extend beyond graphene. Yet, its growth on Au(111) remains debated, with structural phases highly discussed due to the possible incorporation of substrate adatoms into the phosphorus adlayer. Here, we present a general methodology combining noncontact atomic force microscopy (nc-AFM) and force spectroscopy to unambiguously discriminate between competing structural models of BlueP/Au(111) obtained by density functional theory (DFT). Each phosphorus phase is resolved at the atomic scale by nc-AFM imaging, while site-dependent force spectroscopy probes local atomic corrugations within the structure. Comparison with probe-particle simulations using DFT coordinates reveals that all structural phases on Au(111) consist of an assembly of BlueP_9_ or BlueP_16_ units stabilized by Au adatoms. These findings not only solve the long-standing debate over the phosphorus–Au(111) interface but also provide an experimental strategy for identifying atomic structures of epitaxial Xenes.

## Graphical abstract

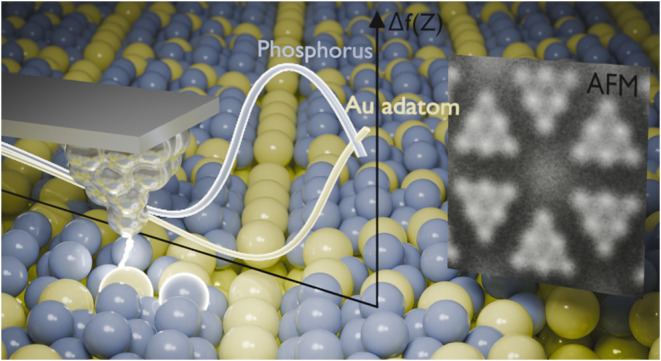

## Introduction

Since the successful exfoliation of a single graphene sheet by Novoselov and Geim,^1^ monoelemental two-dimensional (2D) materials (i.e., Xenes)^2^ are the focal point of tremendous research efforts aimed at revealing new physical, chemical and electronic properties.^3^ Unlike graphene, most Xenes exhibit significant buckling in their honeycomb lattice, arising from their larger covalent radii and mixed sp^2^–sp^3^ hybridization.^4^ This atomic corrugation combined with strong spin–orbit coupling (both absent in graphene) is predicted to offer exciting prospects for tailoring electronic band structures and developing emergent quantum phases such as the quantum anomalous Hall effect^5–7^ or topological superconductivity.^8^ Among them, blue phosphorene (BlueP), a buckled quasi-planar atomic structure of phosphorus atoms is an interesting candidate, which has similar conducting properties as black phosphorus^9^ with a tunable semiconducting band gap depending on the number of layers^10^ and applied external fields.^11^

Similar to most monoelemental Xenes (such as silicene, stanene, borophene and many more), a phosphorus monolayer can be grown by molecular beam epitaxy (MBE) on noble metals such as Au(111) and Cu(111). However, the local bonding environment and hybridization with the substrate have a strong influence on the final structure, leading to the coexistence of multiple phases and potential alloy formation.^12^ Accurately determining atomic structures of these phases using diffraction techniques can be difficult since the data interpretation can be hindered by coexisting structures. Moreover, while the spatial resolution of scanning tunneling microscopy (STM) provides valuable information down to the atomic scale, it usually fails to accurately determine the intrinsic corrugation of the atomic layer as this technique is primarily sensitive to the local density of states (LDOS). To address this, we recently introduced an experimental method that combines low-temperature noncontact atomic force microscopy (nc-AFM) using carbon monoxide (CO) terminated tips^13,14^ with site-dependent force spectroscopy to quantify both the lateral atomic spacing and the out-of-plane corrugation of phosphorus and silicon structures on Ag(111) with sub-Ångström precision.^15,16^ The experimental observations are systematically validated by a comparison with simulations of nc-AFM images and force spectra using the probe-particle model (ppafm) from Hapala et al.^17–19^ This model, which takes as input the atomic coordinates of structures relaxed by density functional theory (DFT), accurately simulates the deflection of the CO molecule attached to the tip, when subject to long- and short-range forces acting between the tip and the last atoms of the probed atomic lattice. Whereas our approach only consider the atomic corrugation, note also that force spectroscopy can identify the chemical nature of surface atoms even in mixed materials from the variation of short-range interaction forces in force–distance spectra.^20,21^ This comparative method has demonstrated to provide a robust approach for discriminating between competing DFT models within epitaxial Xenes,^15,16^ leading to an unambiguous identification of the atomic structures directly from experimental nc-AFM images.

Phosphorus monolayers on Au(111) were intensively characterized by STM, angle-resolved photoemission spectroscopy (ARPES), low energy electron diffraction (LEED), surface X-ray diffraction (SXRD) and DFT calculations,^22–29^ showing a periodic structure with a rhombus unit cell and a lattice parameter of *a* = 14.4 Å as corresponding to a (5 × 5)-Au(111) surface supercell. Based on STM images showing a hexagonal arrangement of triangular motifs, several competing models were debated in the literature. The initial model proposed by Zhang et al.^22^ described a buckled phosphorene layer on Au(111), in which some buckled atoms were arranged in triangular motifs. Although the model did not fully reproduce the STM observations, it has nonetheless been widely reported in the literature as the “*Blue Phosphorene*” structure (BlueP). To better match STM images, Zhang et al.^24^ later proposed to add six extra P atoms onto a phosphorene monolayer (denoted in the following as BlueP + 6P). However, the structural stability of this configuration has been questioned in a subsequent work.^27^

More recently, Zhao et al.^27^ revisited the P/Au(111) systems using neural network (NN) potential combined with DFT. Their theoretical models described all these phosphorus phases on Au(111) by considering small P_18_ (P_32_) BlueP patches stabilized by 9 (12) Au adatoms. Another work by Del Puppo et al.^29^ further provided experimental data by STM, ARPES and SXRD data indicating the incorporation of Au adatoms in the P_18_Au_9_ structure. In the following, we will refer to these Au-BlueP candidate structures as the P_18_Au_9_ and P_32_Au_12_ structures, respectively. Note also that these structures consisting of BlueP patches separated by Au adatoms cannot be considered as pure phosphorene layer in contrast to the initial models of Zhang et al.^22,24^ In fact, the plethora of structural models deduced from identical STM data reflects the inherent difficulty of determining atomic structures in these complex systems, notably because they involve large surface unit cells. In this context, a quantitative characterization by nc-AFM and ppafm at the atomic-scale might provide a definitive discrimination between these competing DFT models.

Here, we employed our nc-AFM based methodology to quantify with sub-Ångström precision the atomic configurations of all phosphorus structures on Au(111) reported in the literature. We systematically compare the experimental nc-AFM images and site-dependent Δ*f*(*Z*) spectroscopy with simulated counterparts using the ppafm with the DFT coordinates as inputs to quantify the atomic spacing and corrugation within each phase. We conclude that all phosphorus phases on Au(111) consist of small BlueP patches stabilized by Au adatoms, thus confirming the P_18_Au_9_ model and contributing to the resolution of the long-standing controversy surrounding the phosphorus-Au(111) interface. Our results demonstrate the importance of this method to unambiguously determine atomic structures of epitaxial Xenes in real-space.

## Results and Discussion

### STM Images of the Phosphorus Structure on Au(111)

Phosphorus atoms were sublimed from a Knudsen cell containing black phosphorus in ultra high vacuum (UHV) onto the Au(111) surface held between 260 and 320 °C (see Methods). Figure 1a shows an STM overview image acquired after deposition of ∼0.5 monolayer (ML). Terraces are covered by two hexagonal patterns, denoted α- and β-phases in Figure 1a, which are resolved with high-resolution in the close-up STM images of Figure 1c,d, respectively. These STM images of repeating triangular motifs are identical to those previously reported in the literature.^23–26,30^ For the α-phase, the lattice of parameter *a*_α_ = 14.3 Å outlined by the black rhombus in Figure 1c corresponds to the (5 × 5) superstructure with respect to Au(111).^24^ The β-phase has also a hexagonal lattice of parameter *a*_β_ = 17.4 Å marked by a dashed rhombus in Figure 1d. Triangles of the α-phase (black in Figure 1c) are composed of three bright protrusions by STM, whereas those in the β-phase exhibits six protrusions (black triangle in Figure 1d). The γ- lattice scales as α- lattice since it corresponds to the addition of P_4_ molecules in the pore of this phase according to ref 26.

**Figure 1:**
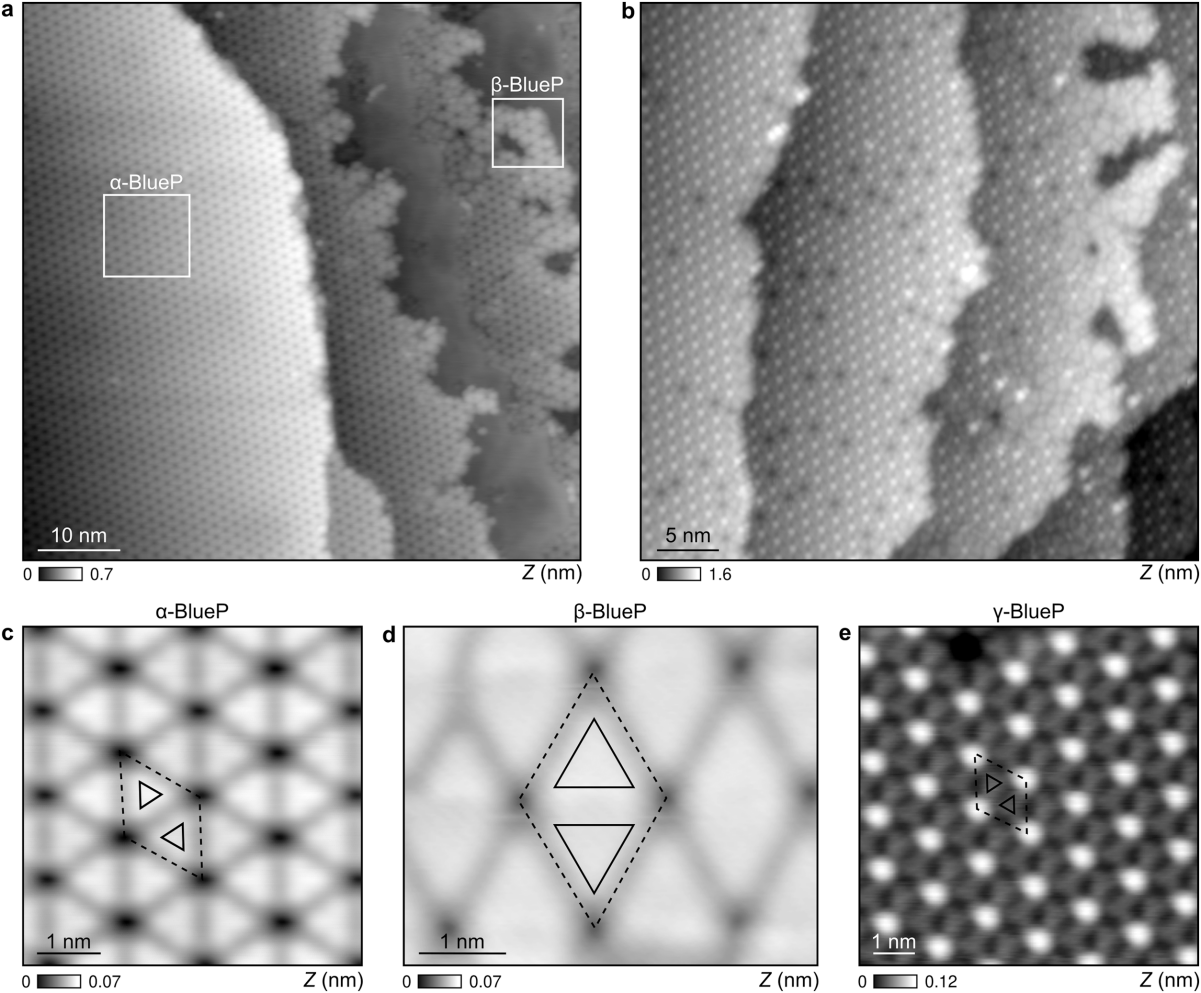
STM images of the phosphorus structures on Au(111). (a, b), Typical STM images of phosphorus domains on Au(111), (*I*_t_ = 10 pA, *V*_s_ = −10 mV). The α- and β-phases, marked on the image, coexist on the surface. (b), STM image of the phosphorus layer on Au(111) after a further deposition of P atoms, leading to the γ- phase (*I*_t_ = 10 pA, *V*_s_ = −10 mV). (c–e), High-resolution STM images of the α- (*I*_t_ = 1 pA, *V*_s_ = −100 mV), β- (*I*_t_ = 1 pA, *V*_s_ = −500 mV) and γ-phase (*I*_t_ = 1 pA, *V*_s_ = −500 mV), respectively. The unit cell of each phosphorus structure is shown by dashed rhombus.

### nc-AFM Image and Force Spectroscopy of the α-Phase

Figure 2a,b show a typical constant-height nc-AFM image with a CO-terminated tip of the α-phase, together with site-dependent Δ*f*(*Z*) spectra (see Methods Section). The nc-AFM image acquired at the position of the STM image of Figure 1c reveals a well-ordered hexagonal lattice of triangular motifs (white triangle), with an unit cell of size 14.3 Å indicated by a dashed rhombus. The close-up nc-AFM images shown in the inset of Figure 2b further estimate the length of the triangle side *l* of about 3.2 Å as well as the gap between neighboring triangles (6.3 Å). Note that low-temperature AFM imaging with CO-terminated tips are performed at constant tip–sample distances (and not at constant–force interaction), which implies that the image only represents the most protruding atoms of the atomically corrugated structure.^15^ The absence of AFM contrast between these triangles does not necessarily mean that no adatoms are present there, but rather that they are further away from the tip due to the variation of atomic corrugation within the structure.

**Figure 2:**
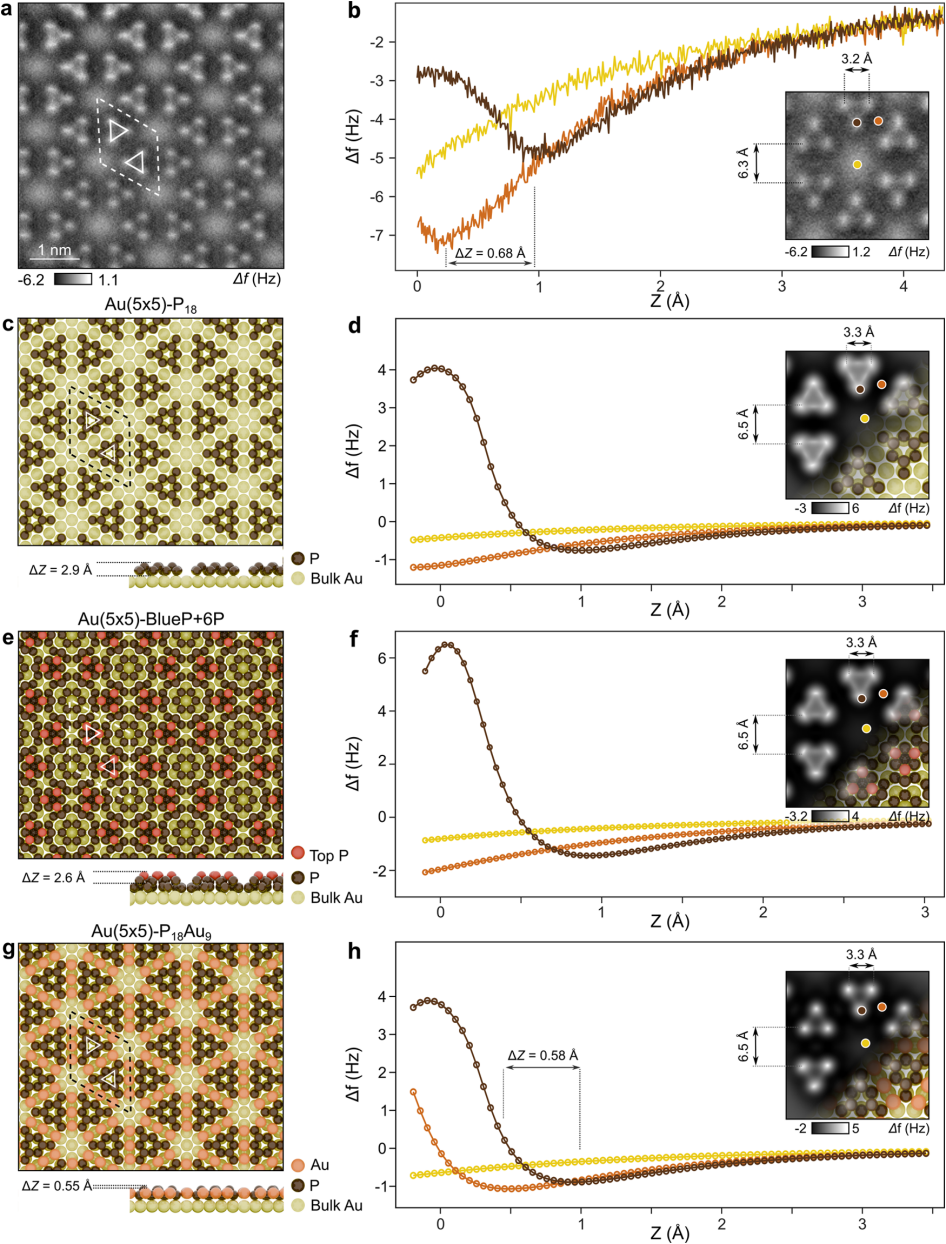
Atomic configuration of the α-phase determined by nc-AFM. (a), Experimental nc-AFM image with a CO-functionalized tip of the α structure, showing a hexagonal pattern of triangular motifs. Each triangle shows three bright protrusion. (b), Δ*f*(*Z*) spectra acquired above the upmost P atoms of a triangle (brown), at the center of the pore (yellow) and in the gap between neighboring triangles (orange). Positions are shown in the nc-AFM image of the inset. The vertical dashed lines indicate the variation Δ*Z* = 0.68 Å between local minima of the brown and orange spectra, associated with the atomic corrugation in the structure. (c, e, g), Top and side views of the Au(5 × 5)-P_18_,^29^ the BlueP + 6P,^24^ and the Au(5 × 5)-P_18_Au_9_ ^27^ DFT models, with P, bulk Au, Au adatoms colored in brown, yellow and orange, respectively. In (e), the red atoms are P atoms adsorbed on top of a phosphorene layer on Au(111). (d, f, h), Simulated Δ*f*(*Z*) spectra using the ppafm at positions shown in the corresponding insets (see Methods Section for the ppafm parameters).

To quantify the atomic corrugation within the structure, we systematically use site-dependent Δ*f*(*Z*) spectroscopic measurements at atomic positions marked in the inset of Figure 2b, i.e., on one atom of the triangle (brown spectra), between the gap of neighboring triangles (orange) and near the center of the pore (yellow). These spectra reflects the force interactions arising between the CO-terminated tip and the front-most P/Au atom. The Δ*f*(*Z*) spectrum for the uppermost atoms (brown) shows a local minimum at *Z* = 0.95 Å, which is related to the repulsive regime of force interaction between tip and sample. Similarly, such local minimum in the Δ*f*(*Z*) curve acquired between neighboring triangles (orange) is also present but shifted to a smaller relative tip–sample distance of *Z* = 0.27 Å. As we previously discussed for silicene on Ag(111),^15^ the difference in vertical position (Δ*Z* = 0.68 Å) between these atoms accounts for the height variation within the structure. However, such a minimum is not visible in the Δ*f*(*Z*) curve of Figure 2 measured near the center of the pore (yellow curve), because the CO tip remains too far from the atom underneath to reach the repulsive force regime. In Supporting Figure 1a, we estimated it to be lower by Δ*Z*_2_ ≈ 1.23 Å as compared to the uppermost P atoms.

To assess the structural models of the α-phase/Au(111), we next compare the experimental data with simulated AFM images and Δ*f*(*Z*) spectra generated by the ppafm using the DFT-relaxed atomic coordinates for each structure (see Methods for the ppafm parameters). Figure 2c shows top and side views of the Au(5 × 5)-P_18_ structure reported by Del Puppo et al.,^29^ with P atoms shown in brown and Au atoms in yellow. The model consists of a hexagonal assembly of small P_9_ patches in registry with the Au(111) substrate, yielding a lattice parameter of 14.3 Å, in agreement with STM measurements. The simulated nc-AFM image (inset of Figure 2d) accurately reproduces the experimental contrast (Figure 2b). Interestingly, although each P_9_ unit is composed of nine phosphorus atoms, the intrinsic buckling of the structure causes only three atoms to protrude upward, while the remaining six P atoms lie closer to the surface. Accordingly, the characteristic signature of a P_9_ BlueP unit in nc-AFM images is a triangle with a side length *l* of 3.3 Å. The (5 × 5)-P_18_ rhombus unit cell aligns along the Au(111) surface directions, leading to a interunit spacing between neighboring P_9_ of 6.5 Å.

By comparing the simulated Δ*f*(*Z*) spectra of Figure 2d with the experimental ones (Figure 2b), we observe a relative agreement for the uppermost P atoms of a P_9_ unit (brown spectra) showing a local minimum followed by a peak of positive Δ*f* values. In contrast, Δ*f*(*Z*) spectra in the void between P_9_ units (orange) and at the center of the pore (yellow) have no such local minimum. This is due to the fact that the tip cannot probe the repulsive force regime in these areas, as a result of the large distance of 2.6 Å between the plane of the uppermost P atoms and the Au plane. Therefore, this structure cannot be validated in light of our nc-AFM measurements.

Figure 2e shows top and side views of the BlueP + 6P structure reported by Zhang et al.^24^ where P and Au atoms are colored in brown and yellow, respectively. Six additional P atoms per unit cell (colored in red) are added on top of the BlueP. The simulated nc-AFM image (inset of Figure 2f) from this model provides the correct side lengths of triangles (*l* = 3.3 Å) as well as the separation of 6.5 Å. However, the Δ*f*(*Z*) spectrum between the *P*_18_ units colored in orange does not show a local minimum, similar to the (5 × 5)-*P*_18_ system, because of the large height difference of 2.6 Å between the uppermost P atoms and the plane of the phosphorene monolayer (see side view of Figure 2e).

We last consider the Au(5 × 5)-P_18_Au_9_ model reported by Zhao et al.^27,28^ and Del Puppo et al.,^29^ which is derived from the Au(5 × 5)-P_18_ structure by incorporating nine additional Au atoms per unit cell positioned between neighboring P_9_ units (orange atoms in Figure 1g). As for the Au(5 × 5)-P_18_ model, the simulated nc-AFM image reproduces well the lateral dimensions of the structure as in the experimental data (inset of Figure 1h), with triangle side lengths *l* = 3.3 Å and interunit separation of 6.5 Å. Importantly, both simulated Δ*f*(*Z*) spectra at the uppermost P atoms (brown) and at the area between P_9_ units (orange) now possess a local minimum marked by dotted lines at 1 Å and 0.42 Å, respectively. The difference in *Z* equals to Δ*Z* = 0.58 Å, which reflects the height difference (0.55 Å) between the uppermost P atoms and the Au adatoms extracted from the DFT coordinates (side view of Figure 2g) and reasonably matches the experimental Δ*Z* of 0.68 Å (Figure 2b).

The relaxed DFT structure of the Au(5 × 5)-P_18_Au_9_ yields a height difference between the uppermost P atoms and the gold substrate within the cavity, Δ*Z*_2_, of 2.6 Å. Therefore, the value estimated from experimental force spectroscopy is approximately a factor of 2 smaller than the DFT prediction (Supporting Figure 1a). Simulations of Δ*f*(*Z*) spectra using the ppafm model (Supporting Figure 1b) can also reproduce a similarly reduced Δ*Z*_2_ value of 1.28 Å when probing near the center of the void. However, we also noticed a strong influence of these minima on the lateral probe position (Supporting Figure 2), resulting in simulated Δ*Z*_2_ values ranging from 1.2 up to 2.59 Å. We therefore attribute the discrepancy between DFT and experiment to strong electrostatic interactions between the surrounding P/Au framework and the macroscopic part of the tip when probing the cavity with a CO-terminated tip. This effect is likely underestimated in the simulations, as the macroscopic shape of the tip is not explicitly taken into account, leading to the observed site-dependent response.

To better resolve the Au adatoms in real-space imaging, Supporting Figure 3a also shows a series of constant-height nc-AFM images for different tip–sample separations Z, which are compared to nc-AFM simulations (Supporting Figure 3c). At large Z, only the uppermost P atoms are visible, forming triangular characteristic of the P_9_ units. At smaller tip–sample separations (*Z* = 0–0.25 Å), faint features appear between these triangles, as indicated by yellow arrows in the nc-AFM and Laplace filtered images in Supporting Figure 3b,c. Comparison with the nc-AFM simulation indicates that these features can be ascribed to the signature of the incorporated Au adatoms. Note that an improved contrast of these atoms may be achieved using nc-AFM imaging with simultaneous current feedback, as previously shown in the case of three-dimensional molecules.^31,32^ This will be addressed in future works.

Finally, given the agreement between theory and experiments regarding nc-AFM images and site-dependent force spectra, we conclude that the (5 × 5)-P_18_Au_9_ model is the most probable of all competing DFT structures for the α-phase. Furthermore, comparing the (5 × 5)-P_18_Au_9_ and (5 × 5)-P_18_ models, we found that the additional Au atoms stabilize the structure, since the adsorption energy per P atom is −0.81 eV/atom without Au_9_ and −1.04 eV/atom with Au_9_.^29^

### nc-AFM Image and Force Spectroscopy of the β-Phase

Using the same experimental methodology, we investigate the second phosphorus structure on Au(111) referred to as the β-phase. As compared to the α-phase (i.e., Au(5 × 5)-P_18_Au_9_), the β-phase is less discussed in the literature because it usually coexists with the α-phase,^22,24^ making its characterization more challenging. Figure 3a,b show a typical constant-height nc-AFM image of the β-BlueP phase acquired at the position of the STM image of Figure 1d and site-dependent Δ*f*(*Z*) spectra. The nc-AFM image shows a hexagonal lattice of triangular motifs with a unit cell of 17.5 Å (dashed rhombus), corresponding to the Au(6 × 6) surface cell (17.4 Å). As compared to the Au(5 × 5)-P_18_Au_9_ structure, the length of the triangle side is about twice larger (*l* = 6.7 Å) whereas the interunit separation is in the same order of magnitude (6.4 Å). In the nc-AFM image, triangles have now six bright protrusions (white triangle), indicating that this BlueP unit contains more atoms than the P_9_ one observed in the same image (circle in Figure 3a). Site-dependent Δ*f*(*Z*) at the uppermost P atoms (brown), in the gap between triangles (orange) and at the center of the pore (yellow) are shown in Figure 3b, respectively. For the uppermost P atoms, the local minimum is at a relative height of *Z* = 1.35 Å whereas it emerges at *Z* = 0.65 Å in the void between triangles, yielding a Δ*Z* value of 0.70 Å between these atomic positions.

**Figure 3:**
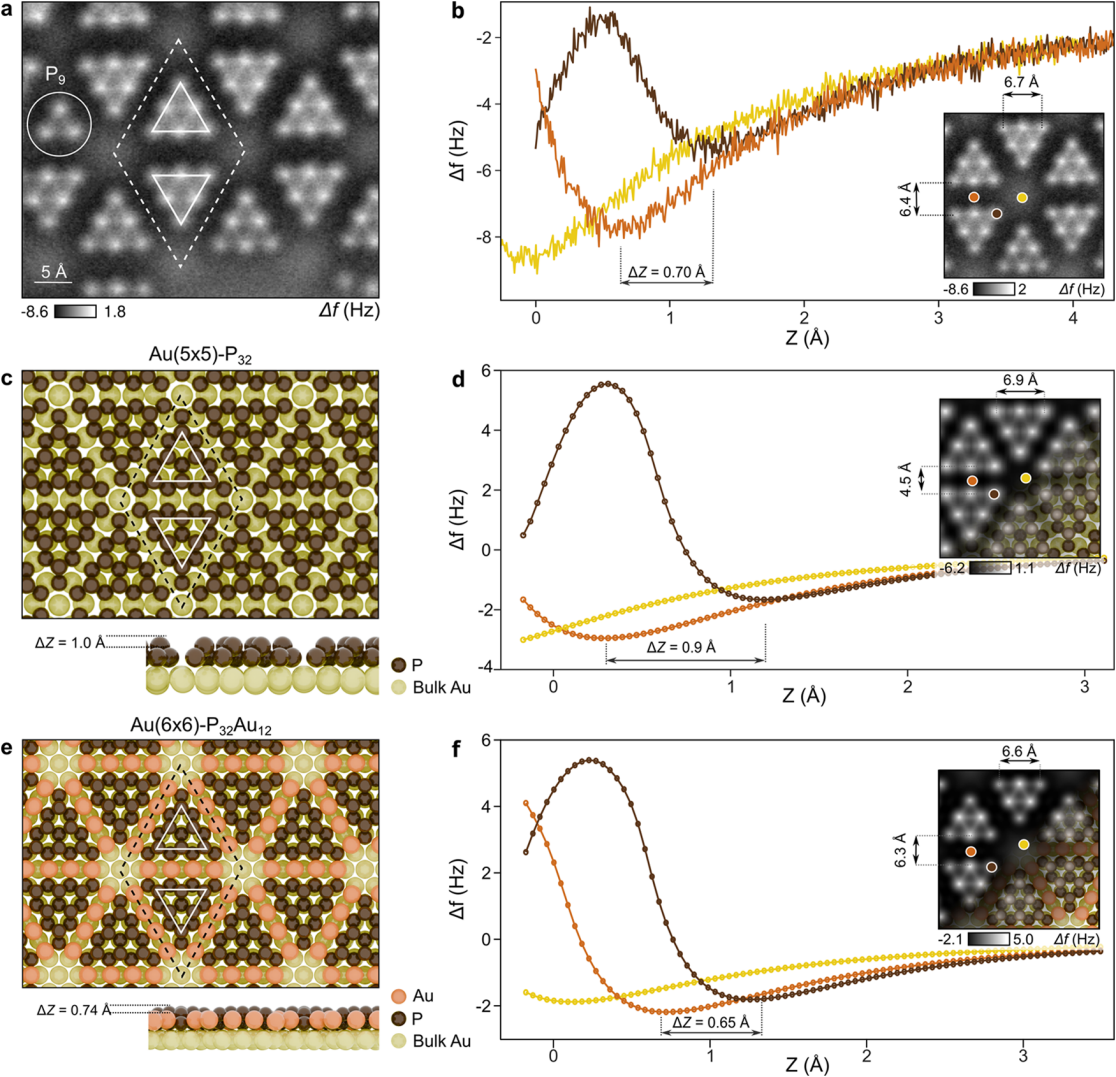
Atomic configuration of the β-phase determined by nc-AFM. (a), Experimental nc-AFM image with a CO-functionalized tip of the β structure, showing a hexagonal pattern of triangular motifs associated with P_16_ units. The white circle indicates one P_9_ unit remaining. (b), Δ*f*(*Z*) spectra acquired above the upmost P atoms of a P_16_ unit (brown), at the center of the pore (yellow) and in the gap between neighboring P_16_ units (orange). Positions are shown in the nc-AFM image of the inset. The vertical dashed lines indicate the variation Δ*Z* = 0.68 Å between local minima of the brown and orange spectra, associated with the atomic corrugation in the structure. (c, e), Top and side views of the Au(5 × 5)-P_32_,^22^ and the Au(6 × 6)-P_32_Au_12_ ^27^ DFT models, with P, bulk Au, Au adatoms colored in brown, yellow and orange, respectively. (d, f), Simulated Δ*f*(*Z*) spectra using the ppafm at positions shown in the corresponding insets (see Methods Section for the ppafm parameters).

Figure 3c presents the top and side views of the DFT model by Zhang et al.,^22^ with P and Au atoms colored in brown and yellow. This structure is a hexagonal assembly of P_16_ units in registry with the Au(5 × 5) surface cell, yielding a lattice parameter of 14.5 Å. Note that this lattice parameter is smaller than our experimental estimate of 17.5 Å. Therefore, the simulated nc-AFM image (inset of Figure 3d) confirms that each P_16_ unit leads to a triangular contrast with six protrusions, whose side lengths are equal to *l* = 6.9 Å. However, the separation between P_16_ units of 4.5 Å is shorter that the experimental one (6.4 Å) because this structure adopts a Au(5 × 5) registry instead of a Au(6 × 6). The simulated Δ*f*(*Z*) spectra shows local minima at both the uppermost P atoms (brown) and in the gap between two P_16_ units (orange). However, the Δ*Z* is about 0.9 Å, which is larger than the experimental one of 0.70 Å (Figure 3b), since it corresponds to the buckling magnitude between the upper and lower P atoms of a P_16_ unit (1.0 Å), as extracted from the DFT model (side view of Figure 3c).

Figure 3e now shows the Au(6 × 6)-P_32_Au_12_ structural model proposed by Zhao et al.,^27,28^ which is composed of P_16_ units separated by Au adatoms in a Au(6 × 6) registry. As a consequence, the simulated nc-AFM image (inset of Figure 3f) now correctly reproduces both the lateral dimensions of the structure, with the triangle side length of *l* = 6.6 Å and the unit separation of 6.3 Å, in close agreement with the experimental nc-AFM image (Figure 3f). The height difference between these atoms is reflected in a Δ*Z* shift of 0.65 Å, similar to the experimental value of 0.70 Å as well as the height variation between these atoms extracted from DFT (0.74 Å). Taken together, these accurate estimates in *X*, *Y* and *Z* directions indicate that the Au(6 × 6)-P_32_Au_12_ model provides the best reproduction of our experimental nc-AFM measurements.

### nc-AFM Image and Force Spectroscopy of the γ-Phase

For the sake of completeness, we also characterize the γ-phase shown by STM in Figure 1e, which is obtained by maintaining the α-phase at about 320 °C during another phosphorus deposition. As this structure directly derives from the α-phase, nc-AFM images again show a hexagonal arrangement of P_9_ units (white triangles), that preserves the Au(5 × 5) registry (dashed rhombus) with additional P clusters occupying the pore sites of the initial Au(5 × 5)-P_18_Au_9_ phase. These clusters appear as bright triangles located at each corner of the dashed rhombus in the nc-AFM image of Figure 4a. Both site-dependent Δ*f*(*Z*) spectra (Figure 4b) acquired at the most protruding atom of a P_9_ unit (brown spectra) and on top of a P cluster (red) show local minima, with a Δ*Z* value of approximately 1.18 Å.

**Figure 4:**
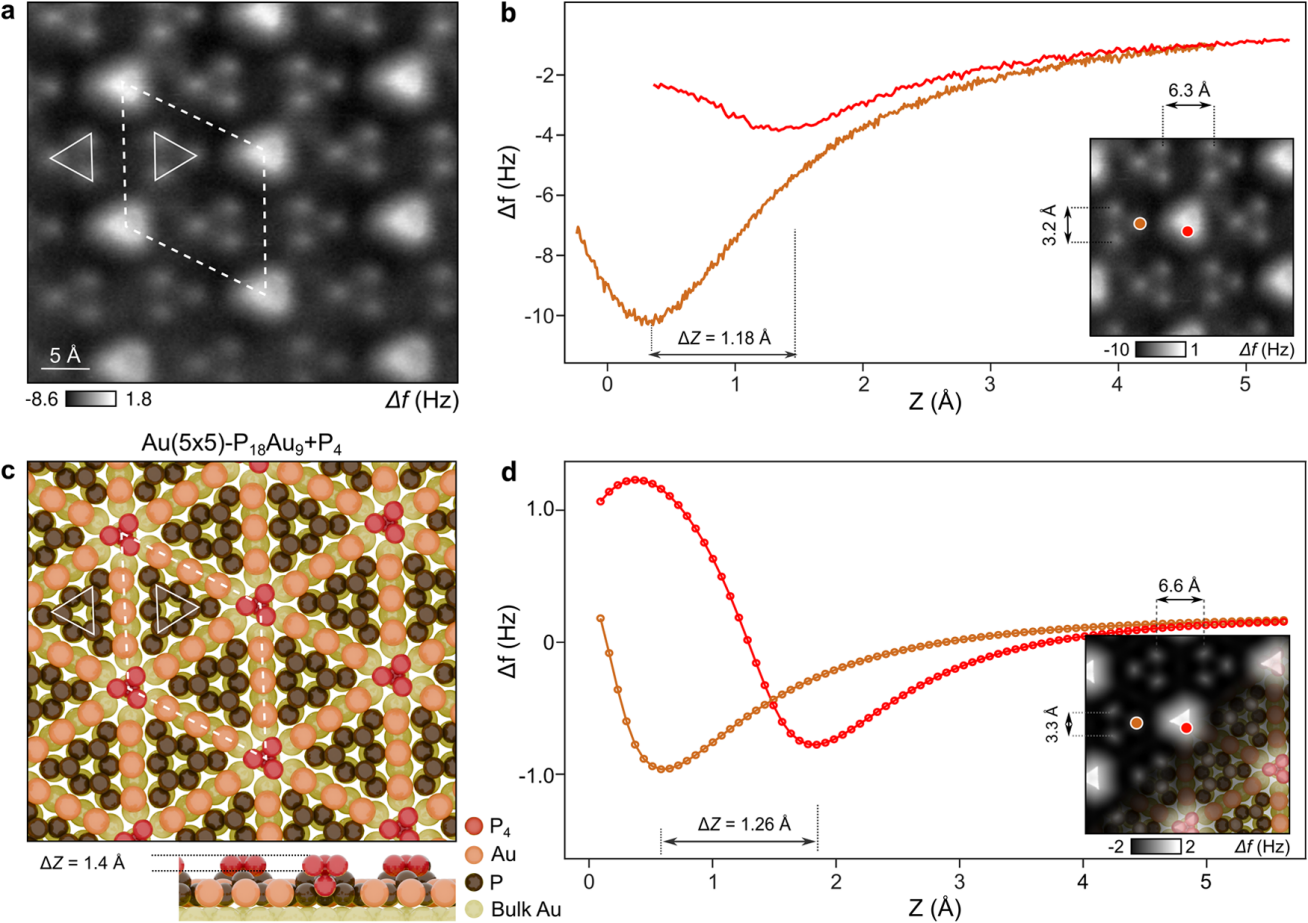
Atomic configuration of the γ-phase determined by nc-AFM. (a), Experimental nc-AFM image with a CO-functionalized tip of the γ structure, showing the hexagonal pattern of the Au(5 × 5)-P_18_Au_9_ structure, together with additional bright triangles located at the corners of the dashed rhombus. (b), Δ*f*(*Z*) spectra acquired above the upmost P atoms of a P_9_ unit (brown) and on top of a bright triangle (red). Positions are shown in the nc-AFM image of the inset. The vertical dashed lines indicate the height difference Δ*Z* = 1.18 Å between local minima of the brown and red spectra associated with the atomic corrugation in the structure. (c), Top and side views of the Au(5 × 5)-P_18_Au_9_ + P_4_ DFT model,^27^ with P, bulk Au, Au adatoms colored in brown, yellow and orange, respectively. Red atoms refer to the P_4_ pyramids, oriented upside down and positioned directly above a central Au atom in the pore. (d), Simulated Δ*f*(*Z*) spectra using the ppafm at positions shown in the corresponding insets (see Methods Section for the ppafm parameters).

Previously, a theoretical model of the γ-phase has been proposed by Zhao et al.^27^ by considering P_4_ pyramidal clusters adsorbed into the pores of the Au(5 × 5)-P_18_Au_9_ structure. Figure 4c reproduces the top and side views of this model denoted Au(5 × 5)-P_18_Au_9_ + P_4_ with P, bulk Au, Au adatoms colored in brown, yellow and orange, respectively. According to ref 27 the P_4_ pyramids shown in red exhibit their most stable adsorption configuration when oriented upside down and positioned directly above a central Au atom of the pore. The simulated nc-AFM image from this structure (inset of Figure 4d) not only perfectly reproduces the triangular contrast from each P_9_ unit, but also the bright features associated with the P_4_ clusters. Note also that the triangular contrast from the P_4_ clusters further corroborates the peculiar upside-down adsorption of the P pyramids. The simulated Δ*f*(*Z*) spectra of Figure 4d extracted at the top of a P_4_ cluster (red) and at the uppermost P atoms of a P_9_ unit (brown) have two local minima, separated by Δ*Z* = 1.26 Å. In comparison to the DFT side view of Figure 4c, the Δ*Z* value is associated with the height difference of 1.4 Å between the P_4_ and P_9_ unit of the structure, which is in agreement with the experimental estimate of Δ*Z* = 1.18 Å. Thus, our experimental results validate the Au(5 × 5)-P_18_Au_9_ + P_4_ configuration as the correct atomic model for the γ-phase.

## Conclusions

Identifying a definitive structural DFT model for the phosphorus interfaces on Au(111) from STM imaging and diffraction techniques has proven both controversial and difficult,^22–26^ particularly due to the unsolved question of whether substrate adatoms might incorporate into the phosphorus adlayers.^27–29^ Furthermore, while DFT formation energies and thermodynamic stability are important metrics, they are often insufficient to resolve structural ambiguities, as the formation of one or another structure can be driven by kinetic pathways rather than the attainment of a global thermodynamic minimum. To address this issue, our work demonstrates a general and robust experimental strategy for identifying the atomic structures of P/Au(111) phases by comparing high-resolution nc-AFM images and site-dependent force spectroscopy with ppafm simulations based on the competing DFT models. For the α-phase, we show that, although STM and nc-AFM images can be reproduced by most DFT models, only complementary force spectroscopic measurements provides an accurate determination of the atomic corrugation in the structure as listed in Table 1 indicating the presence of Au adatoms in the void between neighboring P_9_ units. To date, this key experimental signature can only be described by considering a P_9_ structure stabilized by Au adatoms on the Au(111) substrate (i.e., the Au(6 × 6)-P_18_Au_9_) relative to the others. A similar conclusion is obtained for the β-phase/Au(111) consisting of P_16_ units stabilized by Au adatoms (i.e., the Au(6 × 6)-P_32_Au_12_) as well as the Au(5 × 5)-P_18_Au_9_ + P_4_ structural model.

**Table 1:** Comparison of Lattice Parameters *a*, Atomic Corrugation Δ*Z* and the Side Length *l* of Phosphorus Triangles for Each Phosphorus Phases on Au(111), as Determined by nc-AFM and DFT^a^

		*a*		Δ*Z*		*l*	
Phase	Proposed structures	DFT	nc-AFM	DFT (FS)	nc-AFM	DFT	nc-AFM
α	Au(5 × 5)-P_18_ ^29^	14.4	14.3	2.9 (−)	0.68	3.3	3.2
	Au(5 × 5)-BlueP + 6P^24^	14.4	14.3	2.6 (−)	0.68	3.3	3.2
	Au(5 × 5)-P_18_Au_9_ ^27–29^	14.4	14.3	0.55 (0.6)	0.68	3.3	3.2
β	Au(5 × 5)-P_32_ ^22^	14.4	17.5	1.0 (0.9)	0.70	6.9	6.7
	Au(6 × 6)-P_32_Au_12_ ^27,28^	17.2	17.5	0.74 (0.65)	0.70	6.6	6.7
γ	Au(5 × 5)-P_18_Au_9_ + P_4_ ^27^	14.4	14.3	1.4 (1.26)	1.18	3.3	3.2

a For the DFT values, FS refers to the values extracted from the force spectra simulated using the DFT coordinates. Units are in Ångström.

In their seminal work,^20^ Sugimito et al. demonstrated the chemical identification of individual surface atoms, based on detecting short-range chemical forces between the outermost atom of an AFM tip and the adatoms in force spectroscopic measurements. With this approach, the ratio of maximum attractive forces between pairs of atomic species can serve as fingerprints for chemical recognition. To compare with ppafm simulations, we extracted from our measurements the minimum Δ*f* values of the Δ*f*(*Z*) curves acquired over Au atoms and the uppermost P atoms in both the Au(5 × 5)-P_18_Au_9_ and Au(6 × 6)-P_32_Au_12_ structures, derived from two independent data sets using CO-terminated tips (Figures 2b and 3b). Across both structures under similar experimental conditions, we obtained a ratio of 0.72 between incorporated Au atoms and uppermost P atoms, whereas a value of 0.98 was found when comparing the uppermost P atoms. Extracting the Au/P and P/P relative interaction ratio from the simulated Δ*f*(*Z*) curves (Figures 2h and 3f) yields values of 0.80 and 1.0, respectively, in agreement with the experimental results. In contrast to ref 20 the P/Au(111) systems have much larger atomic corrugation and various atomic bonding,^33^ which may perturb the estimation of this ratio. For instance, for pairs of P atoms within P_9_ units or P_4_ pyramids in the γ-phase, this ratio decreases to 0.4 in the experiments (Figure 4b) and to 0.88 for the simulated spectra (Figure 4d). Despite comparable tip conditions for identical atoms, this discrepancy underscores the strong sensitivity of these ratios to surface corrugation. Future works should focus on this aspect of the force spectroscopic measurements, notably when tackling P alloys or structurally complex P structures.^12^

Establishing confidence in these accurate structural models from experimental nc-AFM data is pivotal for interpreting their electronic properties, guiding improvements in theoretical descriptions, and enabling the controlled synthesis of other low-dimensional phosphorus–metal architectures. Our work significantly advances the fundamental understanding of the P–Au surface chemistry and provides a robust framework for future studies of phosphorus-based nanostructures. It also underscores the broader importance of nc-AFM imaging and local force spectroscopy for distinguishing geometric and electronic effects in the characterization of 2D materials or other Xenes.

## Methods/Experimental Section

### Sample Preparation

The Au(111) substrate, purchased from Mateck GmbH, was cleaned by repeated cycles of Ar^+^ ion sputtering and annealing at 500 °C to remove surface contaminants. Phosphorus atoms were sublimed by heating a black phosphorus crystal contained in a Knudsen effusion cell under ultrahigh vacuum (UHV) conditions. The phosphorus flux was estimated using a quartz crystal microbalance. To obtain the ordered phosphorus structures, the Au(111) substrate was maintained at controlled temperatures during deposition, following the growth conditions described in refs 22–26,29 .

### STM/AFM Experiments

STM experiments were conducted at a temperature of 4.8 K using an Omicron GmbH low-temperature STM/AFM system operated with Nanonis RC5e electronics. All voltages refer to the sample bias *V*_s_ with respect to the tip. AFM measurements were performed with commercially available tuning-fork sensors in the qPlus configuration^34^ equipped with a tungsten tip (*f*_0_ = 26 kHz, *Q* = 10,000 to 25,000), nominal spring constant *k* = 1800, N.m^–1^, oscillation amplitude *A* ≈ 50–100 pm. Constant-height AFM images were obtained in the noncontact mode (frequency-modulated AFM–FMAFM) using tips terminated with a single carbon monoxide (CO) at zero voltage.^13,35^ CO molecules were adsorbed on the sample maintained at low temperature below 20 K. Before its functionalization, the apex was sharpened into the gold surface by gentle indentations. Site-dependent Δ*f*(*Z*) spectroscopic measurements^36^ to determine the atomic corrugation within the phosphorus structures were obtained with CO-terminated tips. All STM/AFM images and Δ*f*(*Z*) spectra of the manuscript are raw data.

### DFT Calculations

All DFT calculations have been performed using the Perdew–Burke–Ernzerhof (PBE) functional^37^ within the generalized gradient approximation (GGA) for exchange-correlation terms and the Grimme-D3 scheme for dispersion corrections.^38^ The Au(111) surface was modeled with periodically repeated 4-layer^27,29^ or 5-layer^24^ slabs with a vacuum spacing ranging from 12 Å^29^ to 20 Å.^27^ k-meshes ranging from 2 × 2 × 1 ^29^ to 5 × 5 × 1 ^27^ were used to sample the first Brillouin zone. The energy cutoffs for the plane wave expansion of the wave functions were ranging from about 15 Ry^24^ (with projector augmented wave method) to 40 Ry.^29^ The convergence thresholds were set to about 10^–4^ Ry/atom^28^ or lower^24,27^ for total energy in self-consistent calculations, and to about 10^–3^ Ry/*a*_0_ for forces on the atoms in the structural optimization cycles. DFT calculations reported from ref 29 were carried out using the Quantum ESPRESSO (QE) package,^39^ while those in refs 24,27,29 with the VASP package.^40^ Further methodological details are reported in ref 29 for the Au(5 × 5)-P_18_ model, in ref 29 for the BlueP + 6P model, in refs 24,27 for the Au(5 × 5)-P_18_Au_9_ model, and in refs 24,27 Au(5 × 5)-P_32_Au_12_ and Au(5 × 5)-P_18_Au_9_ + P_4_ models.

### ppafm Simulations

From the DFT coordinates, we have simulated nc-AFM images and site-dependent Δ*f*(*Z*) spectra with the ppafm 0.4.0 software^17–19^ available on Zenodo.^41^ For the probe settings, we used the Lennard–Jones (LJ) force field with point charge electrostatics (LJ + PC), which only depend on chemical nature of the atoms, and the CO-terminated tip with *Q* = −0.1 e, σ = 0.71 Å and eight multipoles. The CO stiffnesses are set to *K*_*x*_ = *K*_*y*_ = 0.25 N.m^–1^ and *K*_R_ = 30 N.m^–1^ for all simulations of the manuscript. For Supporting Figure 1, we used *K*_*x*_ = 0.1 N.m^–1^, *K*_*y*_ = 0.25 N.m^–1^ and *K*_R_ = 30 N.m^–1^ to produce the distorted nc-AFM contrast at close tip–sample separation. Typical scan settings are 0.1 × 0.1 × 0.04 Å^3^ for the scan steps and 35 × 35 Å^2^ for the scan size at a distance of 6.5 Å with oscillation amplitude of 0.5 Å. The *df* settings used for the Δ*f*(*Z*) simulations and spectra are *k* = 1.8 N.m^–1^, *f*_0_ = 26.2 kHz and *z* steps = 85.

## Supplementary Material



## Data Availability

The data that support the findings of this study are available on the Zenodo repository 10.5281/zenodo.18348381.

## Notes

Height variation Δ*Z*_2_ between the uppermost P atoms and the Au substrate at the center of the void; influence of position within the void on the simulated Δ*Z*_2_ value; and evolution of the nc-AFM contrast with the tip–sample separation (PDF)
