# Supplementary material for: Revealing the Atomic Structure of Blue Phosphorus Phases on Au(111) with Noncontact Atomic Force Microscopy
Source: ACS Nano. 2026 Jun 8;20(24):17423–32. doi: 10.1021/acsnano.6c03130 (PMC13296600; doi:10.1021/acsnano.6c03130)
Supplement: Supplementary file 1 [file nn6c03130_si_001.pdf]

Supporting Information for "Revealing the Atomic Structure of Blue Phosphorus Phases on Au(111) with Non-Contact Atomic Force Microscopy"

Outhmane Chahib,[†] Alberto Verdini,[‡] Zhenyu Li,[¶] Abdelkader Kara,[§] Simone del Puppo,[‖] Maria Peressi,[‖] Ernst Meyer,[*,†] and Rémy Pawlak[*,†]

†*Department of Physics, WSS Research Center for Molecular Quantum Systems, University of Basel, Klingelbergstrasse 82, 4056 Basel, Switzerland*

‡*CNR – Instituto Officina dei Materiali (IOM), S.S. 14 km 163.5 in AREA Science Park, 34129 Trieste, Italy*

¶*State Key Laboratory of Precision and Intelligent Chemistry, University of Science and Technology of China, Hefei 230026, China*

§*Department of Physics, University of Central Florida, Orlando, FL 32816, USA*

‖*Physics Department, University of Trieste, via A. Valerio 2, Trieste 34127, Italy*

E-mail: ernst.meyer@unibas.ch; remy.pawlak@unibas.ch

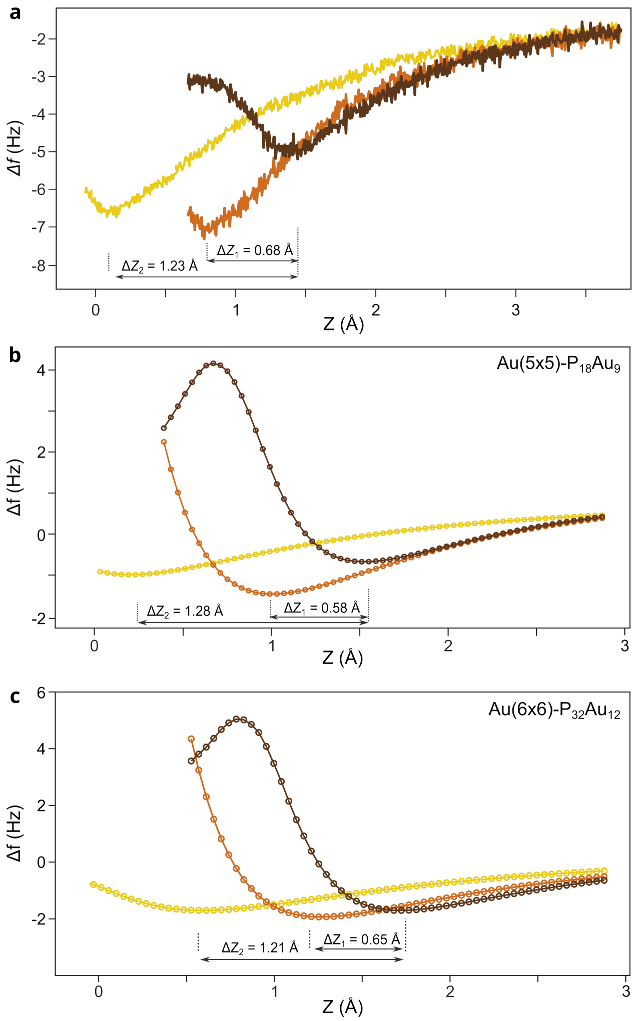

Supporting Figure 1: Height variation ΔZ_2 between the uppermost P atoms and the Au substrate at the center of the void. (a) $\Delta f(Z)$ spectra acquired above the upmost P atoms of a triangle (brown), at the center of the pore (yellow) and in the gap between neighboring triangles (orange). The vertical dashed lines indicate the variation $\Delta Z_1 = 0.68$ Å between local minima of the brown and orange spectra, associated with the atomic corrugation between P and Au adatoms in the Au(5×5)-$P_{18}Au_9$ structure. The variation $\Delta Z_2 = 1.2$ Å between local minima of the brown and yellow spectra refer to the experimental estimate of the height difference between the uppermost P atom and the Au substrate in the void. (b-c), Simulated $\Delta f(Z)$ spectra using the ppafm at a P atom (brown), Au adatom (orange) and Au substrate in the void (yellow) for the Au(5×5)-$P_{18}Au_9$ and Au(6×6)-$P_{32}Au_{12}$, respectively.

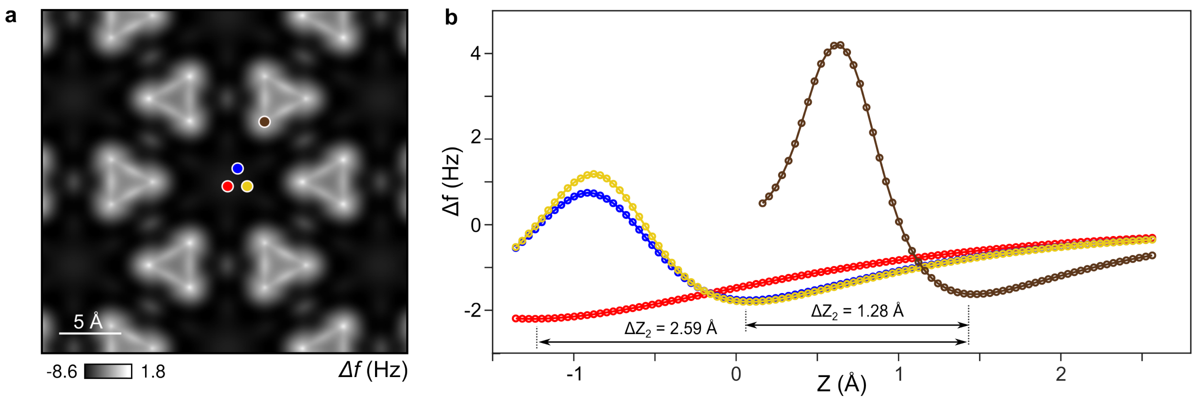

Supporting Figure 2: Influence of position within the void on the ΔZ_2 value. (a) Simulated nc-AFM image using the ppAFM model of the Au(5×5)-$P_{18}Au_9$. (b) Simulated $\Delta f(Z)$ spectra at the P atoms (brown) as compared to three positions into void shown in (a). The estimated $\Delta Z_2 = 1.2$ Å.

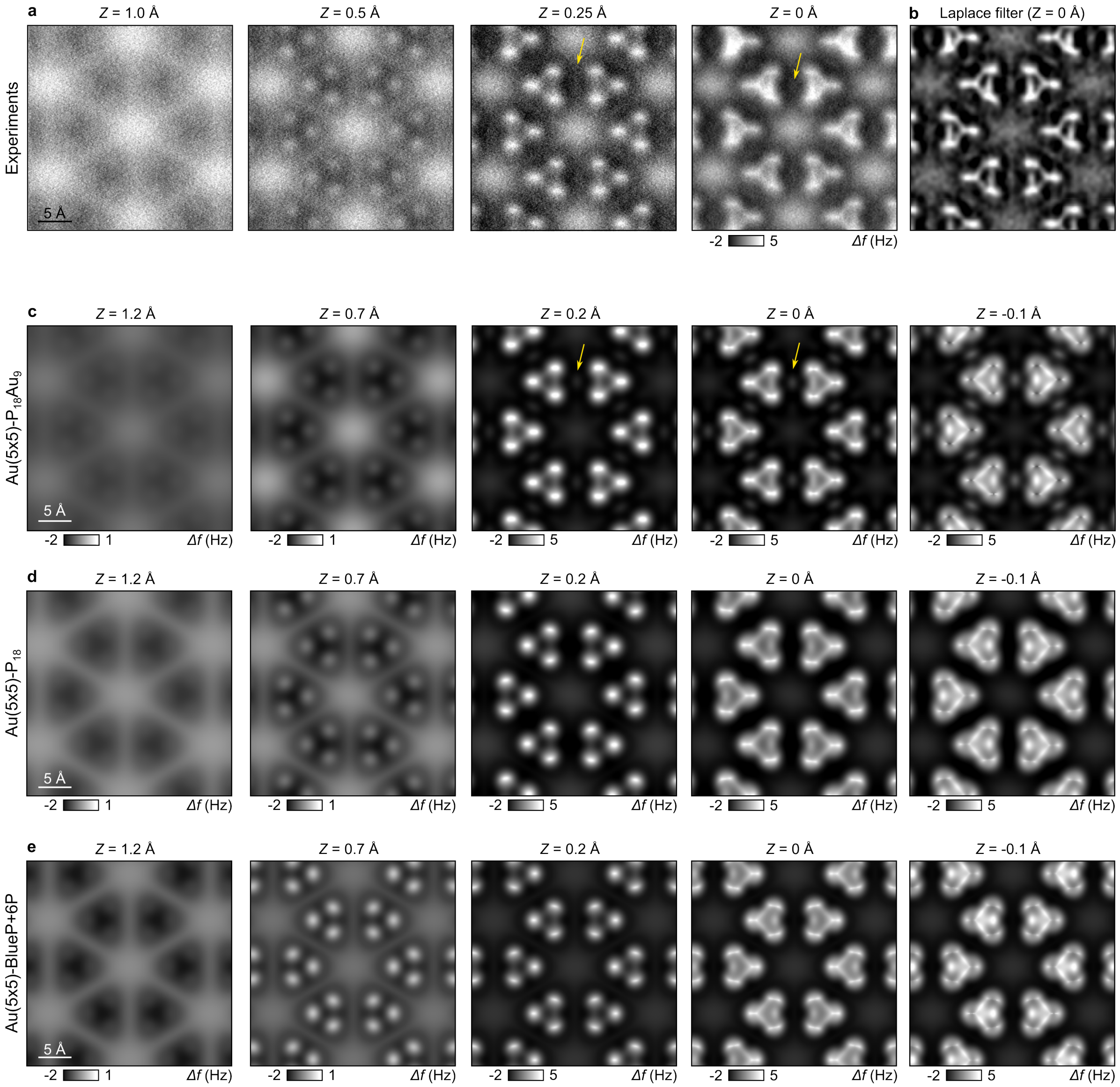

Supporting Figure 3: Evolution of the nc-AFM contrast for the α-phase with the tip-sample separation. **a**, Series of constant-height nc-AFM image for different tip-sample separations Z. **b**, Laplace filtered nc-AFM image at Z = 0 Å. **c-e**, Series of nc-AFM simulation using the ppAFM model for different Z considering the Au(5×5)-$P_{18}Au_9$, the Au(5×5)-P_{18} and the Au(5×5)-BlueP+6P structures, respectively. To best reproduce the distorted contrast at close tip-sample separation (Z = 0 Å), we used $K_x = 0.1$ N.m^{-1}, $K_y = 0.25$ N.m^{-1} and $K_R = 30$ N.m^{-1} (see Methods). The yellow arrows in **a**, **b** and **c** show the signature of the Au adatoms in the nc-AFM image, absent in the case of the other structures.
